# miR‐27a‐3p regulates intestinal cell proliferation and differentiation through Wnt/β‐catenin signalling

**DOI:** 10.1111/cpr.13757

**Published:** 2024-09-27

**Authors:** Chang Li, Yuning Zhou, Yinping Jiang, Zhijie Yin, Heidi L. Weiss, Qingding Wang, B. Mark Evers

**Affiliations:** ^1^ Markey Cancer Center, University of Kentucky Lexington Kentucky USA; ^2^ Department of Surgery University of Kentucky Lexington Kentucky USA

## Abstract

Intestinal stem cells differentiate into absorptive enterocytes, characterised by increased brush border enzymes such as intestinal alkaline phosphatase (IAP), making up the majority (95%) of the terminally differentiated cells in the villus. Loss of integrity of the intestinal epithelium plays a key role in inflammatory diseases and gastrointestinal infection. Here, we show that the intestinal microRNA (miR)‐27a‐3p is an important regulator of intestinal epithelial cell proliferation and enterocyte differentiation. Repression of endogenous miR‐27a‐3p leads to increased enterocyte differentiation and decreased intestinal epithelial cell proliferation in mouse and human small intestinal organoids. Mechanistically, miR‐27a‐3p regulates intestinal cell differentiation and proliferation at least in part through the regulation of retinoic acid receptor α (RXRα), a modulator of Wnt/β‐catenin signalling. Repression of miR‐27a‐3p increases the expression of RXRα and concomitantly, decreases the expression of active β‐catenin and cyclin D1. In contrast, overexpression of miR‐27a‐3p mimic decreases the expression of RXRα and increases the expression of active β‐catenin and cyclin D1. Moreover, overexpression of the miR‐27a‐3p mimic results in impaired enterocyte differentiation and increases intestinal epithelial cell proliferation. These alterations were attenuated or blocked by Wnt inhibition. Our study demonstrates an miR‐27a‐3p/RXRα/Wnt/β‐catenin pathway that is important for the maintenance of enterocyte homeostasis in the small intestine.

## Graphical abstract

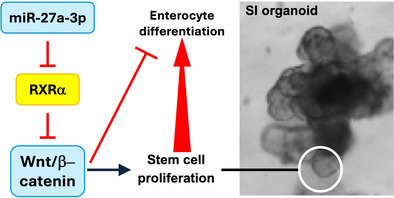

## 1 INTRODUCTION

Mammalian intestinal epithelial cells (IECs) have important physiological functions including absorption of macro‐ and micronutrients from food, secretion of hormones and antimicrobial peptides and regeneration following physical damage.^1^, ^2^ Renewal of IECs occurs approximately every 5–7 days and requires a careful balance between cell proliferation and differentiation to maintain proper lineage ratios and support absorptive, secretory and barrier functions.^3^ Intestinal stem cells, located at the base of the intestinal mucosal crypts, divide and differentiate into specific cell lineages such as absorptive cells. Absorptive enterocytes, characterised by increased brush border enzymes such as intestinal alkaline phosphatase (IAP), make up the majority (95%) of the terminally differentiated cells in the villus. Intestinal protective mechanisms are impaired in patients with inflammatory bowel diseases (IBD) due to lower synthesis and activity of IAP.^4^

MicroRNAs (miRNAs) are small noncoding RNAs consisting of 20–25 bases that regulate the levels of conserved target genes.^5^ miRNAs play an important role in various biological processes, including cell differentiation, proliferation, development and homeostasis.^6^, ^7^ miR‐27a exerts a protective effect on the intestinal mucosa in a dextran sulphate sodium (DSS)—induced colitis mouse model.^8^ Moreover, increased expression of miR‐27a was found in the intestinal tissues of rats with necrotizing enterocolitis compared to the control tissues.^9^

miR‐27a‐3p is significantly increased in faeces of patients with Crohn's disease (CD) compared with healthy controls.^10^ Moreover, miR‐27a‐3p promotes endothelial barrier recovery through Wnt/β‐catenin signalling pathway in mouse astrocytes^11^ and is upregulated in colorectal cancer (CRC) specimens relative to normal colonic mucosa.^12^, ^13^ Moreover, miR‐27a‐3p controls retinoic acid receptor α (RXRα) expression in human hepatoma cells and CRC cells.^12^, ^14^ However, the role of miR‐27a‐3p in intestinal epithelial cell proliferation and differentiation is not known.

The Wnt/β‐catenin signalling pathway plays a central role in intestinal homeostasis. Activation of Wnt/β‐catenin signalling drives stem cell self‐renewal and cell specialization during development as well as adult tissue homeostasis.^15^, ^16^, ^17^ Glycogen synthase kinase 3β (GSK‐3β) phosphorylates β‐catenin in the absence of Wnt and leads to ubiquitin‐mediated proteolysis of β‐catenin.^18^ The binding of Wnt ligands to the Frizzled transmembrane receptors leads to the stabilisation of β‐catenin, which enters the nucleus to activate the transcription of multiple target genes that regulate intestinal epithelial proliferation and differentiation.^19^ Our previous findings identified a critical role of SIRT2/Wnt/β‐catenin and NFAT5/Wnt/β‐catenin signalling in intestinal differentiation and proliferation.^20^, ^21^

In the present study, we demonstrate an important role of miR‐27a‐3p in the maintenance of intestinal cell homeostasis. We show that miR‐27a‐3p regulates intestinal stem cell proliferation and enterocyte differentiation through the regulation of RXRα/Wnt signalling.

## 2 MATERIALS AND METHODS

### 2.1 Mouse intestinal crypt isolation and organoid culture

All animal procedures were approved by the University of Kentucky Institutional Animal Care and Use Committee. Intestinal crypts were isolated as previously described.^20^ Briefly, mouse small intestine (SI) was opened longitudinally, washed with ice‐cold PBS to remove the luminal contents and cut into 2–4 mm pieces. The intestinal fragments were incubated in ice‐cold PBS containing 10 mM EDTA for 60 min at 4°C. Crypts were released by shaking with ice‐cold PBS. Washing in ice‐cold PBS was repeated until most of the crypts were released, as determined by microscopic analysis. Crypt suspensions were passed through a 70 μm cell strainer and centrifuged at 300 × g for 5 min. Isolated crypts were mixed with Matrigel (Corning, Corning, NY, 356231) and cultured in IntestiCult Organoid Growth Medium (Stemcell Technologies, Vancouver, BC, Catalogue # 06005) as described previously.^22^ To determine the role of miR‐27a‐3p mimic in the intestine, mouse SI organoids were infected with lentivirus expressing miR‐27a‐3p mimic (Sigma, St. Louis, MO, MLMIR0237) or miRNA mimic NC (Sigma, NCLMIR002). The infected organoids were cultured in the presence of puromycin (2 μg/mL) to select for organoid cells with stable overexpression of miR‐27a‐3p mimic as described.^23^

### 2.2 Human duodenum epithelial cell isolation and organoid culture

Histologically normal human duodenal samples were obtained from patients at the University of Kentucky undergoing pancreatic resection for cancer. Collection of all patient materials for this study was obtained following patient consent and approved by the University of Kentucky Institutional Review Board (IRB # 48678). Samples from the operating room were immediately placed in ice‐cold PBS. The intestinal fragments were incubated in ice‐cold PBS containing 10 mM EDTA for 60 min at 4°C. After incubation, crypts were released by shaking with ice‐cold PBS. Crypt suspensions were collected and passed through a 100 μm cell strainer and centrifuged at 300 × g for 5 min. Isolated crypts were mixed with Matrigel (Corning) and cultured in IntestiCult Organoid Growth Medium (Human, Stem Cell Technologies, Catalogue #100–0340). To determine the role of miR‐27a‐3p, mouse and human organoids were incubated with either miR‐27a‐3p miRCURY LNA miRNA inhibitor or miRNA negative control oligos (Qiagen, Germantown, MD).

### 2.3 Western blot analysis

Western blotting was performed as we have previously described.^24^ Total protein was resolved on a 10% polyacrylamide gel and transferred to polyvinylidene fluoride membranes. Membranes were incubated for 1 h at room temperature in blotting solution and then incubated in primary antibody diluted in 5% bovine serum albumin solution containing 0.02% sodium azide overnight at 4°C. Antibodies to Cyclin D1 (ab134175) from Abcam, Boston, MA, OLFM4 (#39141), Na,K‐ATPase (#3010 CST), CDX2 (#3977, CST), PCNA (sc‐56, Santa Cruz, Dallas, TX), VILLIN (sc‐7672, Santa Cruz), β‐catenin (610,154, BD Transduction Laboratories, Franklin Lakes, NJ), active β‐catenin (#8814 CST) and β‐actin (A1978, Sigma) were used and following blotting with a horseradish peroxidase‐conjugated secondary antibody, protein expression was visualised using an enhanced chemiluminescence (ECL) detection system.

### 2.4 Quantitative real time RT‐PCR analysis

Total RNA was extracted using a RNeasy Mini Kit (Qiagen) and treated with DNase (RQ1, Promega, Madison, WI). Synthesis of cDNA was performed using reagents in the TaqMan Reverse Transcription Reagents Kit (Applied Biosystems, Foster City, CA). TaqMan probe and primers for human and mouse IAP, FABP1, VILLIN, KRT20, Na,K‐ATPase, SI, CDX2, LGR5, ASCL2, OLFM4 and GAPDH were purchased from Thermo Fisher Scientific (Waltham, MA). All values were normalised to GAPDH levels. To determine the expression of mature miRNA species, total RNA was extracted using a mirVana miRNA Isolation Kit (Thermo Fisher Scientific). cDNA for miRNA gene expression analysis was synthesised using a TaqMan Advanced miRNA cDNA Synthesis Kit (Thermo Fisher Scientific). hsa‐miR‐27a‐3p Assay ID: 478384 mir, mm‐miR‐27a‐3p Assay ID: mmu478384_mirand U6 snRNA Assay ID: 001973 purchased from Thermo Fisher Scientific. For miRNA expression levels, all values were normalised to U6 levels. Quantitative real time RT‐PCR analysis was performed with an Applied Biosystems Prism 7000HT Sequence Detection System using TaqMan universal PCR master mix as we have described previously.^20^, ^25^

### 2.5 IF and IHC staining

Organoids grown in Matrigel were harvested in cold phosphate‐buffered saline (PBS) followed by centrifugation at 300 × g for 5 min. Organoids were fixed with 4% formaldehyde at room temperature for 1 h, centrifuged at 300 × g for 5 min to remove the 4% formaldehyde and resuspended in HISTOGEL (Thermo Scientific; HG‐4000‐012). Paraffin‐embedded organoid sections were processed for routine immunofluorescence (IF) or immunohistochemistry (IHC) staining. DAPI (Electron Microscopy Sciences, Hatfield, PA; 17,989–20) was used as a counterstain to detect nuclei. Images were acquired with a confocal microscope (Nikon ECLIPSE Ti2) at 40× magnification. IHC staining was performed as we have described previously.^26^ Tissues were processed for routine IF or IHC staining using the following antibodies: anti‐KRT20 (Abcam; ab854), anti‐Na,K‐ATPase (Cell Signalling; #3010), anti‐VILLIN (Santa Cruz Biotechnology, sc‐58,897) and anti‐FABP1 (Cell Signalling Technology, Danvers, MA; #13368) and cyclin D1 (Abcam; ab134175). Negative controls (including no primary antibody or isotype‐matched mouse immunoglobulin G) were used in each assessment. Cyclin D1‐positive cells were identified and quantitated using the HALO image analysis platform. Image contrast was adjusted for visualisation purposes and quantification was always applied on raw, non‐adjusted images.

### 2.6 Alkaline phosphatase activity assay

Organoids grown in Matrigel were harvested in cold PBS followed by centrifugation. Organoid cell lysates were used to determine IAP activity by a commercially available kit (Sigma, AP0100) as we have described.^21^, ^27^

### 2.7 Statistical Analysis

Bar graphs were generated to represent mean ± SD for each cell culture condition. Relative levels of mRNA and IAP activity were calculated based on mean levels in the non‐targeting control (NC) group. Statistical tests were performed using two‐sample t‐test for comparisons between NTC versus miR‐27a‐3p inhibitor or analysis of variance with contrast statements for pairwise testing of multiple groups of NC, miR‐27a‐3p+/‐XAV mimics. Multiple testing was addressed using adjusted *p*‐values from the Holm's method. Homoscedasticity of variance assumptions for the parametric tests were assessed and appropriate data transformation was employed if indicated. *p*‐values <0.05 were considered statistically significant.

## 3 RESULTS

### 3.1 Repression of miR‐27a‐3p promotes intestinal enterocyte differentiation in mouse intestinal organoids

**FIGURE 1:**
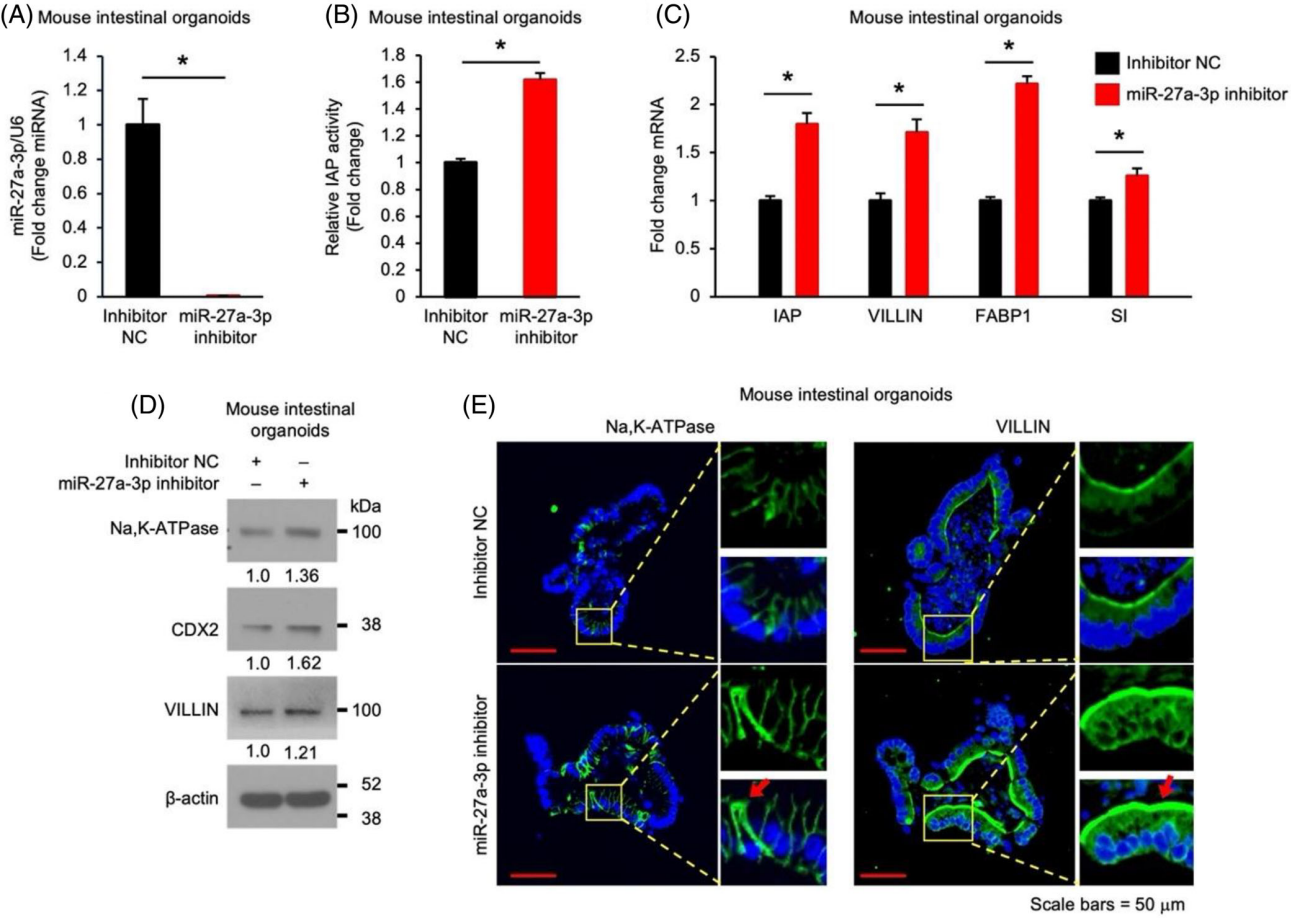
Repression of miR‐27a‐3p promotes intestinal enterocyte differentiation in mouse intestinal organoids. Mouse jejunum crypts were isolated and organoids were cultured with either the miR‐27a‐3p inhibitor (0.75 μM) or inhibitor non‐targeting control (NC) for 5 d. Total miRNA, RNA and protein were extracted for analysis. (A) Expression of endogenous miR‐27a‐3p was significantly decreased by the miR‐27a‐3p inhibitor (*n* = 3; data represent mean ± SD). (B) Inhibition of miR‐27a‐3p increases intestinal alkaline phosphatase (IAP) activity in mouse organoids (*n* = 3; data represent mean ± SD). (C, D) Inhibition of miR‐27a‐3p increases the expression of enterocyte markers. The expression of enterocyte markers was analysed by qPCR (C) and western blot (D) (*n* = 3; data represent mean ± SD). (E) The enterocyte markers, Na,K‐ATPase and VILLIN, were analysed by immunofluorescence. The images of Na,K‐ATPase (left) and VILLIN (right) were taken with a Nikon confocal microscope. Boxed areas are magnified on the right. Red arrows indicate the cells with higher expression of Na,K‐ATPase or VILLIN; Blue: DAPI; Green: Na,K‐ATPase or VILLIN. Scale bars = 50 μm.

Elevated miR‐27a expression has been found in the inflamed intestinal mucosa,^8^, ^9^ however, the role of miR‐27a in the maintenance of intestinal homeostasis is not known. We have determined the expression pattern of miR‐27a‐3p in mouse intestine. A gradient increase in miR‐27a‐3p levels was found in the intestinal crypts isolated from duodenum, jejunum and ileum (Figure S1).To determine the role of miR‐27a‐3p in intestinal IECs, mouse small intestinal (SI) organoids were incubated with inhibitor NC oligos or miR‐27a‐3p inhibitor to repress endogenous miR‐27a‐3p and intestinal differentiation was assessed. Incubation of IECs with miR‐27a‐3p inhibitor repressed the endogenous miR‐27a‐3p expression as expected (Figure 1A). Importantly, treatment with the miR‐27 inhibitor significantly increased intestinal enterocyte differentiation as noted by increased IAP activity (Figure 1B) and mRNA expression of enterocyte markers IAP, VILLIN, FABP1 and sucrase‐isomaltase (Figure 1C). The increased protein expression of VILLIN and other enterocyte markers (e.g., Na,K‐ATPase and CDX2) was demonstrated by western blotting (Figure 1D) and IF staining (Figure 1E). These results show that repression of miR‐27a‐3p promotes intestinal enterocyte differentiation, which suggests an important role of miR‐27a‐3p in the maintenance of intestinal homeostasis.

Figure S1 Expression pattern of miR‐27a‐3p in mouse small intestinal mucosa. Mouse small intestinal crypts were isolated and total miRNA was extracted. Expression of miR‐27a‐3p was determined by qPCR. Expression was normalised to U6 expression. *n* = 4 mice.

### 3.2 miR‐27a‐3p repression decreases stem cell proliferation in mouse intestinal organoids

**FIGURE 2:**
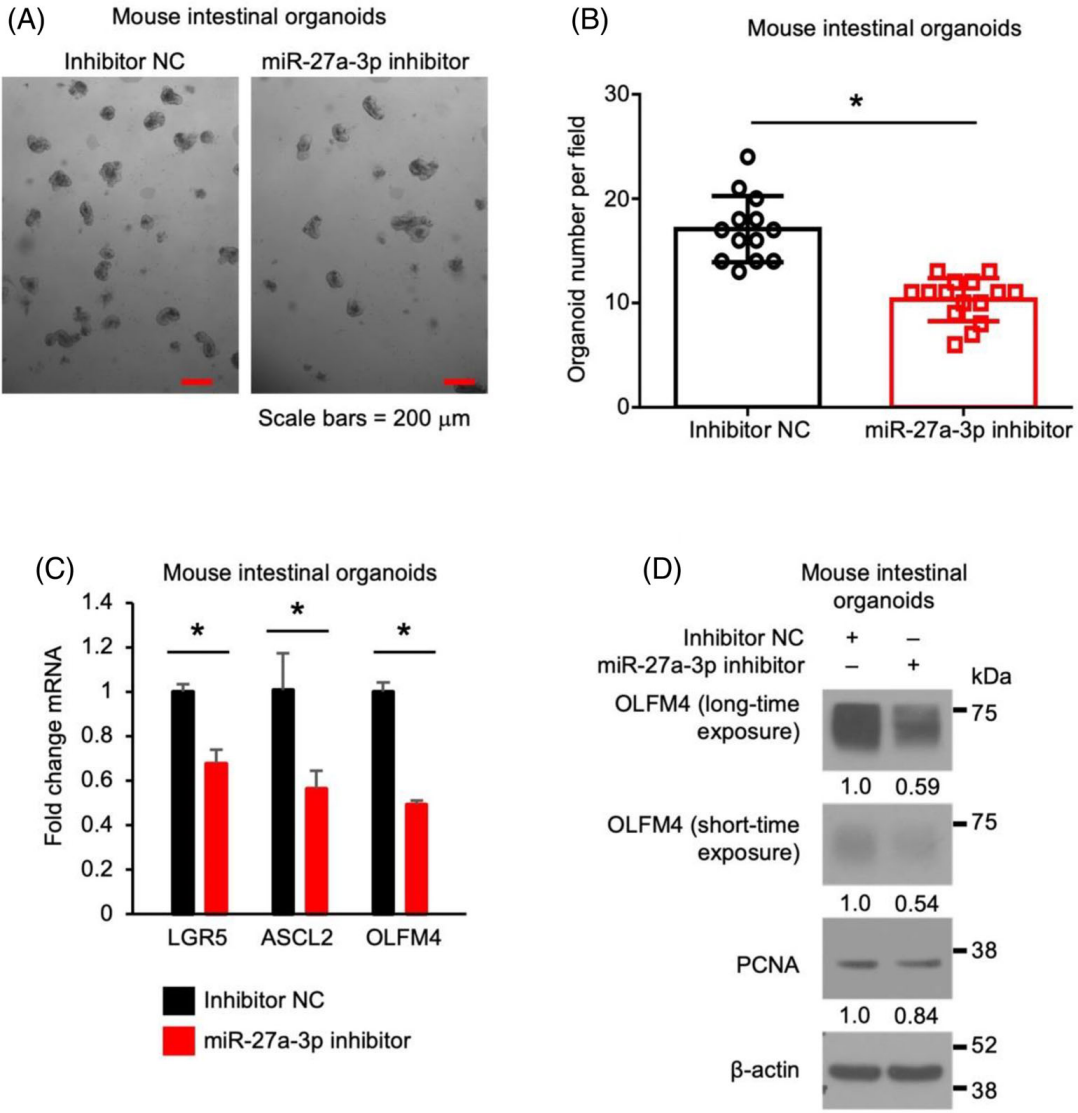
miR‐27a‐3p repression decreases stem cell proliferation in mouse intestinal organoids. Mouse jejunum organoids were incubated with miR‐27a‐3p (0.75 mM) inhibitor or inhibitor non‐targeting control (NC) for 3 d. (A) Morphology of organoids incubated with inhibitor NC or miR‐27a‐3p inhibitor. (B) Organoid numbers per field (*n* = 13 fields per NC group, *n* = 15 fields per miR‐27a‐3p inhibitor group) were analysed. (C, D) Expression of the stem cell markers (LGR5, ASCL2 and OLFM4) and proliferation marker (PCNA) were analysed by qPCR (C) and western blot (D), respectively (*n* = 3; data represent mean ± SD).

To determine the effects of miR‐27a‐3p on intestinal stem cell proliferation, mouse SI organoids were incubated with miR‐27a‐3p inhibitor. As shown in Figure 2, miR‐27a‐3p inhibitor repressed organoid growth as noted by decreased organoid formation (Figure 2A,B). Moreover, miR‐27a‐3p repression decreased the expression of stem cell markers LGR5, ASCL2 and OLFM4 mRNA (Figure 2C). Decreased expression of OLFM4 and proliferating marker PCNA was demonstrated by western blot (Figure 2D). These results demonstrate that miR‐27a‐3p is required for the maintenance of stem cell proliferation.

### 3.3 Repression of miR‐27a‐3p promotes enterocyte differentiation and inhibits stem cell proliferation in human duodenum organoids

**FIGURE 3:**
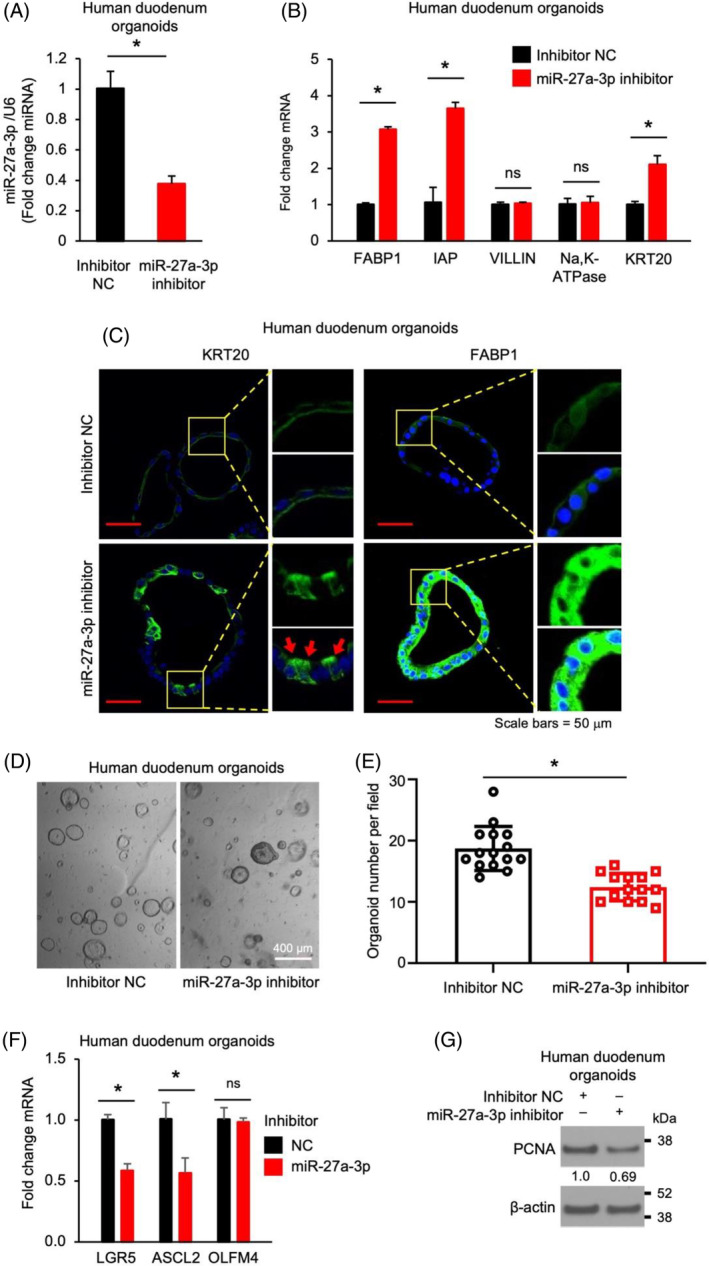
miR‐27a‐3p inhibitor promotes enterocytes differentiation and inhibits stem cell proliferation in human duodenum organoids. The human duodenum organoids were incubated with 1 μM inhibitor non‐targeting control (NC) or the miR‐27a‐3p inhibitor for 5 d. miRNA and RNA were harvested for qPCR analysis. (A) Incubation with the miR‐27a‐3p inhibitor significantly decreased the expression of endogenous miR‐27a‐3p in human duodenum organoids (*n* = 3; data represent mean ± SD). (B) The enterocyte differentiation markers were analysed by qPCR (*n* = 3; data represent mean ± SD). (C) The differentiation markers, KRT20 and FABP1, were analysed by IF. The images of KRT20 (left) and FABP1 (right) were taken by Nikon confocal microscopy. Boxed areas are magnified on the right. Red arrows indicate the cells with higher expression of KRT20; Blue: DAPI; Green: KRT20 or FABP1. Scale bars = 50 μm. (D) Morphology of organoids incubated with inhibitor NC or miR‐27a‐3p inhibitor. (E) Organoid numbers per field (*n* = 15 fields per NC group, *n* = 14 fields per miR‐27a‐3p inhibitor group) were analysed. (F) The stem cells markers (LGR5, ASCL2 and OLFM4) were determined by qPCR (*n* = 3; data represent mean ± SD). (G) The protein level of the PCNA was determined by western blot.

We have shown an important role of miR‐27a‐3p in mouse IEC proliferation and differentiation. To determine whether this occurs in human IECs, we incubated human duodenum organoids with the miR‐27a‐3p inhibitor. Treatment with miR‐27a‐3p inhibitor significantly repressed endogenous miR‐27a‐3p expression in human IECs as expected (Figure 3A). Concomitantly, miR‐27a‐3p inhibitor increased enterocyte maturation as noted by the increased expression of enterocyte markers FABP1, IAP and KRT20 mRNA (Figure 3B). Increased KRT20 and FABP1 protein expression was also demonstrated by IF analysis (Figure 3C). Similar to mouse IECs, miR‐27a‐3p inhibitor repressed organoid growth as noted by decreased organoid formation (Figure 3D,E). Moreover, miR‐27a‐3p repression resulted in decreased expression of intestinal stem cell markers LGR5 and ASCL2 detected by qPCR (Figure 3F) and decreased protein expression of proliferating marker PCNA determined by western blotting (Figure 3G). These data demonstrate a general role for miR‐27a‐3p in the regulation of proliferation and differentiation in human and mouse IECs.

### 3.4 miR‐27a‐3p mimic inhibits enterocytes differentiation in mouse organoids

**FIGURE 4:**
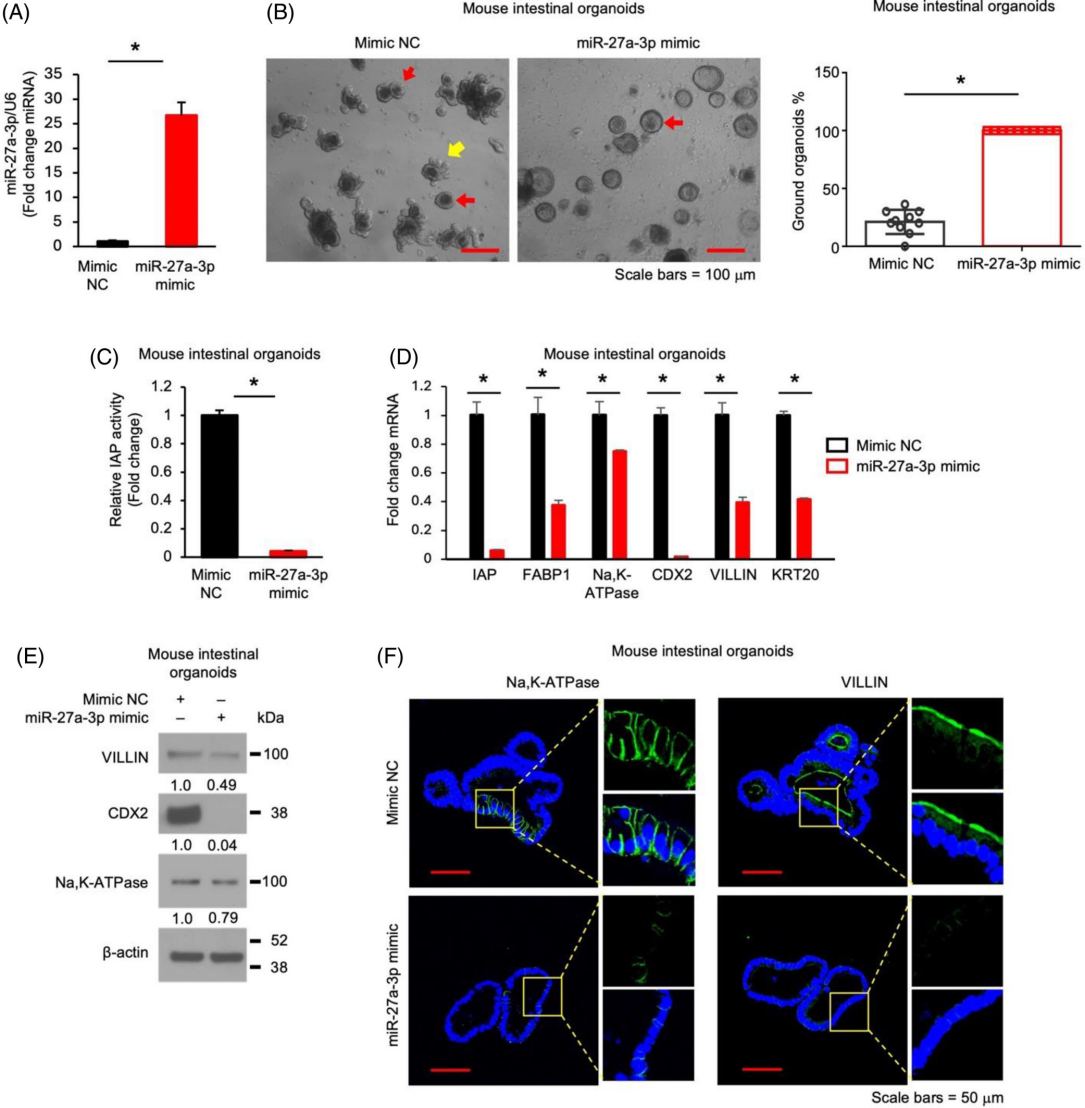
miR‐27a‐3p mimic inhibits enterocyte differentiation in mouse organoids. Mouse jejunum organoids were infected with lentivirus vectors expressing either mimic non‐targeting control (NC) or the miR‐27a‐3p mimic and selected using puromycin. (A) The overexpression of miR‐27a‐3p was confirmed in organoids with stable miR‐27a‐3p mimic and overexpression was determined by qPCR (*n* = 3; data represent mean ± SD). (B) Morphologic analysis of organoids shows that organoids are rounder in response to miR‐27a‐3p mimic overexpression. The images were randomly taken from organoids with either mimic NC or the miR‐27a‐3p mimic. A representative image is shown in the left panel. The red arrow indicates the round organoids; the yellow arrow indicates the budding organoids. Quantification of the round organoids is shown in the right panel; *n* = 10 fields. (C) Overexpression of the miR‐27a‐3p mimic decreases intestinal alkaline phosphatase (IAP) activity (*n* = 3; data represent mean ± SD). (D, E). Overexpression of the miR‐27a‐3p mimic decreases enterocyte marker expression. The differentiation markers were detected by qPCR (D) and western blot (E), respectively (*n* = 3; data represent mean ± SD). (F) IF analysis of the differentiation markers Na,K‐ATPase and VILLIN. The images of Na,K‐ATPase (left panel) and VILLIN (right panel) were taken with Nikon confocal microscopy. Boxed areas are magnified on the right. Blue: DAPI; Green: Na,K‐ATPase or VILLIN. Scale bars = 50 μm.

We have shown the effects of miR‐27a‐3p inhibitor on IECs. To further demonstrate the role of miR‐27a‐3p, we used miR‐27a‐3p mimics. Mouse SI organoids were infected with lentivirus vector expressing miR‐27a‐3p mimic or mimic NC oligo and selected with puromycin. Lentivirus vector‐mediated miR‐27a‐3p mimic overexpression was confirmed (Figure 4A). In contrast to miR‐27a‐3p repression, overexpression of miR‐27a‐3p increased numbers of organoids with round morphology (Figure 4B), indicating an increased stemness.^28^ In addition, overexpression of miR‐27a‐3p repressed enterocyte differentiation as noted by decreased IAP activity (Figure 4C) and decreased expression of enterocyte markers as determined by qPCR (Figure 4D), western blotting (Figure 4E) and IF staining (Figure 4F). Together, our findings further confirm an important role of miR‐27a‐3p in the regulation of IEC proliferation and differentiation.

### 3.5 miR‐27a‐3p regulates RXRα/β‐catenin signalling

**FIGURE 5:**
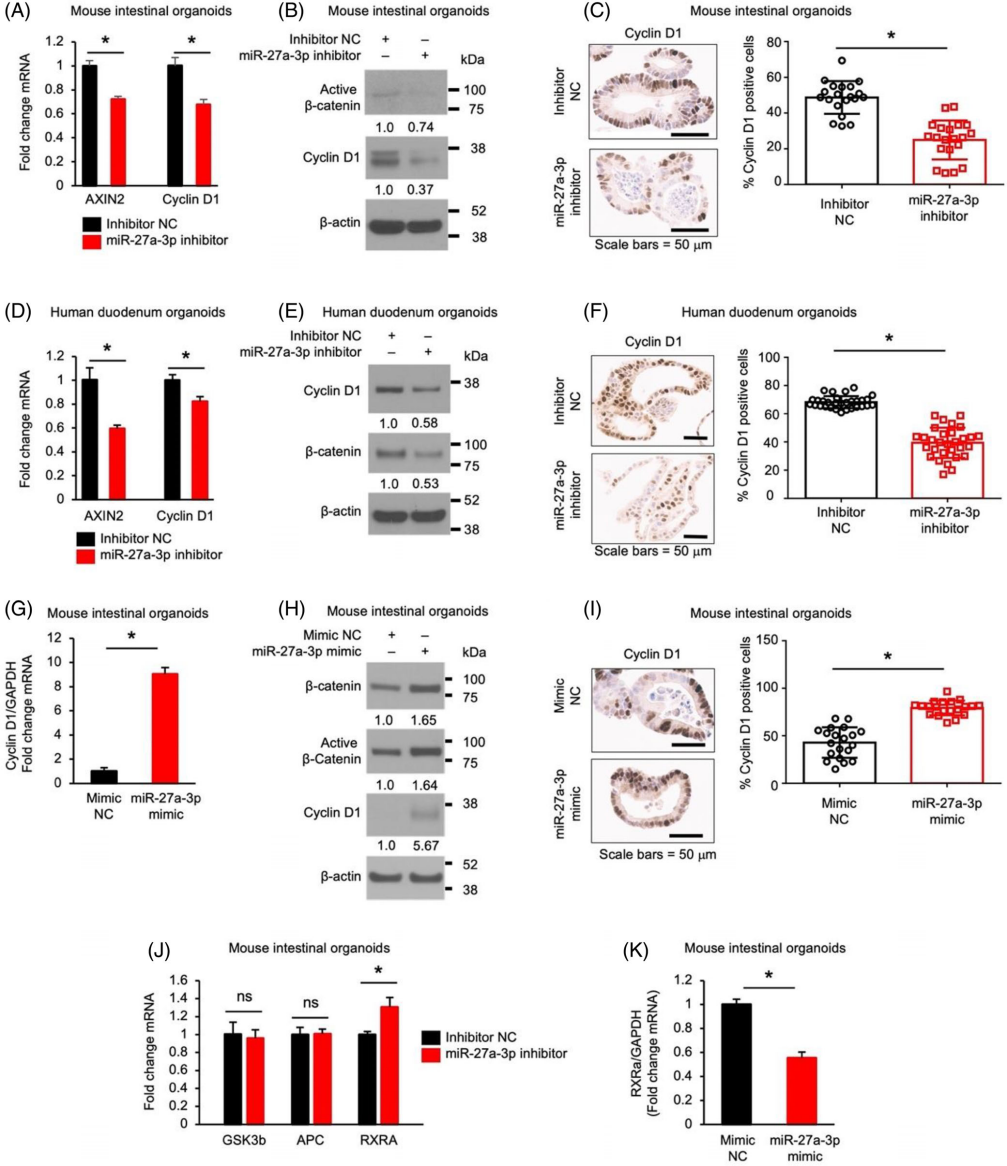
miR‐27a‐3p regulates RXRα/β‐catenin signalling. Mouse intestinal organoids were incubated with inhibitor non‐targeting control (NC) or the miR‐27a‐3p inhibitor for 5 d. (A) The expression of Wnt/β‐catenin targets (AXIN2 and cyclin D1) was determined by qPCR (*n* = 3; data represent mean ± SD). (B) The expression of active β‐catenin and cyclin D1 was determined by western blot. (C) The expression of cyclin D1 was analysed by IHC. A representative image is shown in the left panel. Quantification of cyclin D1 positive cells was performed using HALO software and is shown in the right panel; *n* = 20 organoids. (D–F) miR‐27a‐3p repression decreases the expression of Wnt pathway target genes in human duodenum organoids. Human duodenum organoids were incubated with either inhibitor NC or the miR‐27a‐3p inhibitor for 5 d. qPCR analysis of AXIN2 and cyclin D1 expression (D) (*n* = 3; data represent mean ± SD). Western blot analysis of cyclin D1 and β‐catenin expression (E). IHC analysis of cyclin D1 expression (F). A representative image is shown in the left panel; cyclin D1 positive cells were analysed by HALO software and are shown in the right panel; *n* = 30 organoids. (G–I) Overexpression of the miR‐27a‐3p mimic activates Wnt/β‐catenin signalling in mouse intestinal organoids. The expression of cyclin D1 was detected by qPCR (G) (*n* = 3; data represent mean ± SD). The protein levels of β‐catenin, active β‐catenin and cyclin D1 were determined by western blot (H). Cyclin D1 positive cells were analysed by IHC (I); *n* = 20 organoids. (J) Mouse intestinal organoids were treated with either inhibitor NC or the miR‐27a‐3p inhibitor for 5 d. Expression of GSK3β, APC and RXRα was determined by qPCR (*n* = 3; data represent mean ± SD). (K) Overexpression of the miR‐27a‐3p mimic decreases RXRα mRNA levels determined by qPCR (*n* = 3; data represent mean ± SD).

miR‐27a‐3p promotes Wnt/β‐catenin signalling pathway in mouse astrocytes.^11^ To determine whether miR‐27a‐3p regulates intestinal cell proliferation and differentiation through the Wnt/β‐catenin pathway, we first determined the effects of miR‐27a‐3p on Wnt signalling in normal IECs. Treatment with the miR‐27a‐3p inhibitor repressed Wnt signalling as shown by the decreased expression of Wnt target genes AXIN2 and cyclin D1 (Figure 5A) and decreased active (non‐phosphorylated) β‐catenin^29^ (Figure 5B). miR‐27a‐3p repression also decreased cyclin D1 positive cells as demonstrated by IHC staining followed by HALO analysis (Figure 5C). miR‐27a‐3p repression in human IECs decreased the expression of AXIN2 and cyclin D1 mRNA (Figure 5D), decreased the expression of cyclin D1 and β‐catenin protein (Figure 5E) and decreased cyclin D1 positive cells (Figure 5F). Consistently, overexpression of the miR‐27a‐3p mimic increased cyclin D1 mRNA expression (Figure 5G) accompanied by the increased total and active β‐catenin and cyclin D1 protein expression (Figure 5H) and cyclin D1 positive cell numbers (Figure 5I) in mouse SI organoids. Together, these data demonstrate that miR‐27a‐3p activates Wnt/β‐catenin in both mouse and human IECs.

miR‐27a‐3p has been shown to repress the expression of GSK3β,^30^ adenomatous polyposis coli (APC),^31^ and RXRα^12^ in certain cell types, which inactivate Wnt/β‐catenin signalling.^12^, ^32^, ^33^ To determine how miR‐27a‐3p impacts Wnt/β‐catenin signalling, the effects of miR‐27a‐3p on the expression of GSK3β, APC and RXRα were determined. Treatment with miR‐27a‐3p inhibitor increased RXRα expression without affecting the expression of GSK3β and APC in mouse SI organoids (Figure 5J). In contrast, overexpression of the miR‐27a‐3p mimic significantly decreased RXRα expression in mouse IECs (Figure 5K) suggesting that miR‐27a‐3p activates Wnt/β‐catenin signalling through the regulation of RXRα expression in IECs.

### 3.6 miR‐27a‐3p regulates intestinal enterocyte differentiation through the Wnt/β‐catenin pathway

**FIGURE 6:**
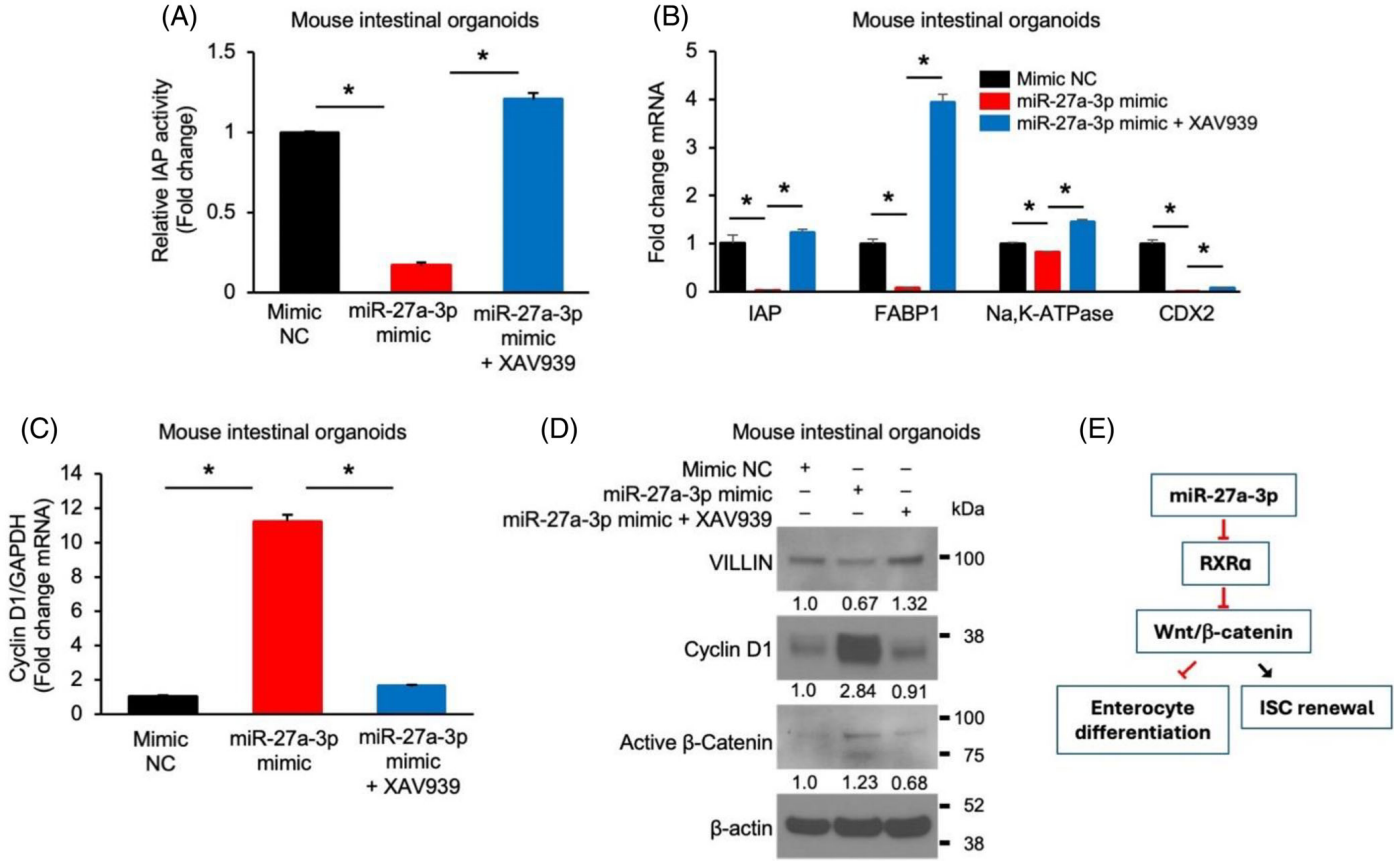
miR‐27a‐3p regulates intestinal enterocyte differentiation through the Wnt/β‐catenin pathway. Mouse intestinal organoids with stable overexpression of mimic non‐targeting control (NC) or the miR‐27a‐3p mimic were cultured for 2 d followed by incubation with or without Wnt inhibitor XAV939 (10 μM) for an additional 3 d. (A) Intestinal alkaline phosphatase (IAP) activity was determined. Treatment with XAV939 reverses the decrease of IAP activity induced by overexpression of the miR‐27a‐3p mimic (*n* = 3; data represent mean ± SD). (B, C) The expression of enterocyte differentiation markers (B) and the Wnt target cyclin D1 (C) was analysed by qPCR (*n* = 3; data represent mean ± SD). (D) The protein levels of VILLIN, cyclin D1 and active β‐catenin were determined by western blot. (E) Summary model illustrating the proposed role of miR‐27a‐3p/RXRα/Wnt/β‐catenin signalling pathway in intestinal epithelial cell proliferation and differentiation.

Wnt/β‐catenin pathway plays a crucial role in IEC proliferation and differentiation; therefore, we next determined whether miR‐27a‐3p regulated IEC proliferation and differentiation through the regulation of Wnt/β‐catenin signalling. Mouse SI organoids infected with lentivirus expressing miR‐27a‐3p mimic or miRNA mimic NC were incubated with or without XAV939, which inhibits Wnt signalling.^34^ As shown in Figure 6, overexpression of the miR‐27a‐3p mimic decreased enterocyte differentiation, as shown by decreased IAP activity (Figure 6A) and expression of IAP, FABP1, Na,K‐ATPase and CDX2 (Figure 6B). These decreases were attenuated or blocked by combination treatment with XAV939. In contrast, treatment with the miR‐27a‐3p mimic increased the expression of cyclin D1 mRNA (Figure 6C) and protein (Figure 6D) and decreased VILLIN protein and increased active β‐catenin expression (Figure 6D). These alterations were blocked by treatment with XAV939. Together, our results demonstrate that miR‐27a‐3p regulates IEC proliferation and differentiation through the regulation of Wnt/β‐catenin signalling (Figure 6E).

## 4 DISCUSSION

The balance between intestinal stem cell renewal and differentiation is crucial for the maintenance of intestinal homeostasis. miR‐27a‐3p has been noted to be upregulated in intestinal tissues and faeces in response to inflammation.^9^, ^10^ Moreover, miR‐27a‐3p has been demonstrated to exert a protective effect on the intestinal mucosa in DSS‐induced colitis.^8^ In this study, we provide evidence showing that miR‐27a‐3p regulates IEC proliferation, differentiation and stemness by targeting RXRα and activating Wnt/β‐catenin signalling. Importantly, our findings suggest a novel role for miR‐27a‐3p/RXRα/Wnt/β‐catenin pathway in the maintenance of intestinal epithelial homeostasis.

The importance of miRNA‐based regulation of proliferation and differentiation is only beginning to be understood in IECs.^35^ Using mouse and human intestinal organoid models, we uncovered a novel role for miR‐27a‐3p in IECs. We show that miR‐27a‐3p promotes IEC proliferation and inhibits enterocyte differentiation. Our findings strongly support the notion that miR‐27a‐3p is essential for maintenance of normal intestinal homeostasis. miR‐27a‐3p has been shown to play an important role in the regulation of proliferation and differentiation in other types of cells.^36^, ^37^, ^38^, ^39^, ^40^, ^41^ In agreement with our findings, repression of miR‐27a‐3p resulted in increased adipocyte differentiation.^41^ Overexpression of miR‐27a‐3p inhibited, whereas miR‐27a‐3p repression enhanced, the differentiation of lung fibroblasts into myofibroblasts.^36^, ^37^ Moreover, miR‐27a‐3p could directly inhibit differentiation in goat skeletal muscle satellite cells.^38^ In contrast, miR‐27a‐3p promotes osteogenic differentiation^39^ and erythroid differentiation.^40^ Together, these results suggest a selective role for miR‐27a‐3p in different cell types and organs.

miR‐27a‐3p is known to activate Wnt/β‐catenin in CRC cells.^12^ However, the effects of miR‐27a‐3p on Wnt/β‐catenin signalling in normal intestinal cells are not known. We showed that miR‐27a‐3p activates Wnt/β‐catenin signalling in IECs. We found that repression of miR‐27a‐3p increased, while overexpression of miR‐27a‐3p mimics decreased, the expression of active β‐catenin. Wnt activation blocks differentiation and drives hyperproliferation in the intestine.^42^ In contrast, inhibition of Wnt/β‐catenin signalling blocked proliferation and increased enterocyte differentiation.^43^ In agreement with miR‐27a‐3p activation of Wnt/β‐catenin, repression of miR‐27a‐3p results in decreased proliferation and increased enterocyte differentiation, whereas a miR‐27a‐3p mimic increased intestinal cell stemness and decreased enterocyte differentiation. Moreover, the Wnt/β‐catenin signalling is hyperactivated and plays a critical role for CRC growth.^44^ Activation of Wnt/β‐catenin signalling activation has predominantly resulted from the aberrant elevation of β‐catenin due to the loss of negative regulators.^45^ Consistently, upregulated miR‐27a‐3p was found in CRCs.^12^, ^13^ Together, these results indicate an important role for miR‐27a‐3p in regulation of Wnt/β‐catenin signalling in not only CRC cells but also in normal IECs. Importantly, our results suggest that miR‐27a‐3p regulates intestinal cell enterocyte differentiation through regulation of Wnt/β‐catenin signalling.

In certain cell types, miR‐27a‐3p has been shown to repress the expression of GSK3β,^30^ APC,^31^ and RXRα,^12^ which inactivate Wnt/β‐catenin signalling.^12^, ^32^, ^33^ Our results show that repression of miR‐27a‐3p increases the expression of RXRα but not GSK3β and APC, suggesting miR‐27a‐3p activates Wnt/β‐catenin signalling through the regulation of RXRα expression in IECs. RAR‐dependent signalling is important for maintaining the balance of cell types between differentiated enterocytes and undifferentiated progenitors.^46^, ^47^ RAR‐activation restricts proliferation^47^ and increases enterocyte differentiation,^46^ while inhibition of RAR‐dependent signalling inhibits enterocyte differentiation and improves intestinal regeneration.^46^ Together, our results demonstrate that RXRα, acting downstream of miR‐27a‐3p, mediates the effects of miR‐27a‐3p in the regulation of Wnt/β‐catenin signalling and enterocyte differentiation. We show that the miR‐27a‐3p/RXRα axis regulates the Wnt/β‐catenin signalling pathway and enterocyte differentiation. However, since miR‐27‐3p might have multiple downstream targets in IECs, additional effort is needed to explore other possible signalling pathways that are regulated by miR‐27a‐3p in the regulation of proliferation and differentiation in IECs.

The intestinal epithelium of IBD patients undergoes repeated cycles of injury and repair in response to chronic, recurring inflammation.^48^ miR‐27a‐3p has been demonstrated to exert a protective effect on the intestinal mucosa in DSS‐induced colitis.^8^ In addition, we show that miR‐27a‐3p promotes Wnt/β‐catenin pathway activation and intestinal stem cell proliferation. The Wnt signalling pathway is critical for IEC proliferation and renewal of intestinal epithelial stem cells; hyperactivation of this pathway is strongly associated with colon cancer in which miR‐27a‐3p is upregulated.^12^ Since increased miR‐27a‐3p expression is noted in intestinal colitis,^9^, ^10^ our data suggest that miR‐27a‐3p contributes to the maintenance of intestinal homeostasis by promoting IEC proliferation after injury, potentially by enhanced Wnt signalling and stem cell proliferation.

In summary, we have demonstrated a previously unknown regulatory miRNA network that is important for maintaining intestinal epithelial homeostasis and linking miR‐27a‐3p, RXRα and the Wnt/β‐catenin signalling in IECs. Considering the importance of miR‐27a‐3p in intestinal colitis and CRC, our findings contribute to the fundamental understanding of the pathophysiological role of miR‐27a‐3p in the process of IBD as well as in CRC formation and progression.

## AUTHOR CONTRIBUTIONS

Conceptualization, Qingding Wang and B. Mark Evers; methodology, Chang Li, Yuning Zhou, Yinping Jiang, Zhijie Yin, Qingding Wang and B. Mark Evers; formal analysis, Chang Li, Yuning Zhou, Yinping Jiang, Zhijie Yin, Heidi L. Weiss, Qingding Wang and B. Mark Evers; investigation, Chang Li, Yuning Zhou, Yinping Jiang, Zhijie Yin, Qingding Wang and B. Mark Evers; writing, Qingding Wang and B. Mark Evers; supervision, Qingding Wang and B. Mark Evers. All authors have read and agreed to the published version of the manuscript.

## FUNDING INFORMATION

This work was supported by National Institutes of Health grants R01 DK48498 (BME) and R01 CA272669 (QW and BME).

## CONFLICT OF INTEREST STATEMENT

The authors declare no conflict of interest.

## PATIENT CONSENT STATEMENT

All human materials for this study were obtained following patient consent.

## Supporting information


**Figure S1.** Supporting information.

## Data Availability

The data that support the findings of this study are available from the corresponding author upon reasonable request.
