# Supplementary material for: miR‐27a‐3p regulates intestinal cell proliferation and differentiation through Wnt/β‐catenin signalling
Source: Cell Prolif. 2024 Sep 27;58(2):e13757. doi: 10.1111/cpr.13757 (PMC11839187; doi:10.1111/cpr.13757)
Supplement: Supplementary file 1 — Figure S1. Supporting information. [file CPR-58-e13757-s001.docx]

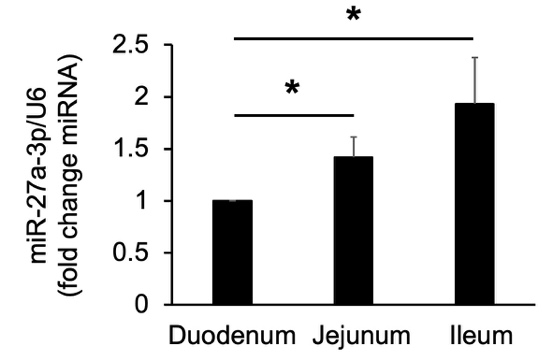


**Figure S1. Expression pattern of miR-27a-3p in mouse small intestinal mucosa.** Mouse small intestinal crypts were isolated and total miRNA was extracted. Expression of miR-27a-3p was determined by qPCR. Expression was normalized to U6 expression. n = 4 mice.
